# Performance Optimization of Priority Assisted CSMA/CA Mechanism of 802.15.6 under Saturation Regime

**DOI:** 10.3390/s16091421

**Published:** 2016-09-02

**Authors:** Mustafa Shakir, Obaid Ur Rehman, Mudassir Rahim, Nabil Alrajeh, Zahoor Ali Khan, Mahmood Ashraf Khan, Iftikhar Azim Niaz, Nadeem Javaid

**Affiliations:** 1 COMSATS Institute of Information Technology, Islamabad 44000, Pakistan; mustafa.shakir@comsats.edu.pk (M.S.); obaid.rehman@comsats.edu.pk (O.U.R.); muddasir1994@yahoo.com (M.R.); mahmoodashraf@comsats.edu.pk (M.A.K.); ianiaz@comsats.edu.pk (I.A.N.); 2 Biomedical Technology Department, College of Applied Medical Sciences, King Saud University, Riyadh 11633, Saudi Arabia; nabil@ksu.edu.sa; 3 CIS, Higher Colleges of Technology, Fujairah, P.O. Box 4114, United Arab Emirates; 4 Department of Engineering Maths and Internetworking, Dalhousie University, Halifax, NS B3J 4R2, Canada; zahoor.khan@dal.ca

**Keywords:** IEEE 802.15.6, WBAN, CSMA/CA, node prioritization, energy efficiency, network lifetime

## Abstract

Due to the recent development in the field of Wireless Sensor Networks (WSNs), the Wireless Body Area Networks (WBANs) have become a major area of interest for the developers and researchers. Human body exhibits postural mobility due to which distance variation occurs and the status of connections amongst sensors change time to time. One of the major requirements of WBAN is to prolong the network lifetime without compromising on other performance measures, i.e., delay, throughput and bandwidth efficiency. Node prioritization is one of the possible solutions to obtain optimum performance in WBAN. IEEE 802.15.6 CSMA/CA standard splits the nodes with different user priorities based on Contention Window (CW) size. Smaller CW size is assigned to higher priority nodes. This standard helps to reduce delay, however, it is not energy efficient. In this paper, we propose a hybrid node prioritization scheme based on IEEE 802.15.6 CSMA/CA to reduce energy consumption and maximize network lifetime. In this scheme, optimum performance is achieved by node prioritization based on CW size as well as power in respective user priority. Our proposed scheme reduces the average back off time for channel access due to CW based prioritization. Additionally, power based prioritization for a respective user priority helps to minimize required number of retransmissions. Furthermore, we also compare our scheme with IEEE 802.15.6 CSMA/CA standard (CW assisted node prioritization) and power assisted node prioritization under postural mobility in WBAN. Mathematical expressions are derived to determine the accurate analytical model for throughput, delay, bandwidth efficiency, energy consumption and life time for each node prioritization scheme. With the intention of analytical model validation, we have performed the simulations in OMNET++/MIXIM framework. Analytical and simulation results show that our proposed hybrid node prioritization scheme outperforms other node prioritization schemes in terms of average network delay, average throughput, average bandwidth efficiency and network lifetime.

## 1. Introduction

Nowadays, smart healthcare services are one of the most preferable demands of our society. A healthcare monitoring service is efficient enough to observe and check a patient anytime and anywhere. As a consequence, plenty of research has been carried out in the last decade by both industry and academia. Numerous state of the art infrastructure and communication technologies for WBAN are investigated in [1]. Bluetooth is one of the widely used communication technologies which aggregates data coming from WBAN. This technology is extensively implemented in devices such as laptops and smart phones. LifeGuard [2] and MagIc [3] are bluetooth enabled healthcare systems. On the other hand, RFID and zigbee or combination of all these technologies can be deployed efficiently for WBAN. Various healthcare systems such as, MEDISN [4] and CodeBlue [5] use IEEE 802.15.4 standard [6] as a communication technology. IEEE 802.15.4 standard is widely used in industrial applications, Internet of Things (IOTs) and smart grids. However, it is not enough to support high data rates for medical applications. A new low power consumption and high data rates supportive standard is required which is suitable for WBANs. The IEEE 802.15 working task group has recently launched the IEEE 802.15.6 standard [7] solely targeting WBAN.

A WBAN is a special purpose sensor network composed of low power, short range and variable data rates sensors planted in or on the body of a patient. These sensors collect real time data of patients in order to treat various diseases such as neurological disorder, asthma, gastrointestinal tract, cancer, and myocardial infection [8].

WBAN deals with two types of traffic namely: emergency traffic and normal traffic. The first type of traffic has a restriction for delay tolerance, i.e., real time data monitoring like ECG. Routine data monitoring like body temperature, blood pressure are the examples of normal data traffic. A WBAN may incorporate a Narrow Band (NB) (400 MHz or 2.4 GHz) together with the Ultra Wide Band (UWB) technology to cover different environments. For some medical applications such as multi-channel EEG and ECG monitoring, it is necessary to use a UWB based WBAN to handle emergency and normal traffic. In NB communication, traffic prioritization is one of the possible solutions to tackle with both types of traffic.

An efficient MAC protocol is one of the suitable vehicles to provide reliable and Quality of Service (QoS) oriented communication. MAC protocols are categorized into contention based and schedule based protocols. Contention based protocols such as CSMA/CA effectively handle the scalability problem of a network. All the nodes simultaneously attempt to access the channel for data transmission. Contention free protocols, i.e., Time Division Multiple Access (TDMA) is responsible for assigning designated time slot to each user. A survey on these two types of protocols for WBAN is presented in [9].

IEEE 802.15.4 standard and IEEE 802.15.6 standard are widely used for research purposes in WBAN. The CSMA/CA mechanism is quite similar in both standards however, IEEE 802.15.6 CSMA/CA increases its Contention Window (CW) size after an unsuccessful medium access. Furthermore, nodes are prioritized by assigning different CW sizes. Hence, IEEE 802.15.6 standard is more suitable for medical data transmission.

A lot of work has already been done on the CSMA/CA mechanism of IEEE 802.15.6 standard.

Authors in [10] evaluate the performance of IEEE 802.15.6 based CSMA/CA MAC protocol for stable network with Immediate ACK policy (I-ACK) and without ACK policy on same CW size (without user priority) and derive mathematical expressions for network delay, throughput and bandwidth efficiency.

In [11] authors derived analytical expressions for energy consumption of CSMA/CA based contention oriented random access scheme and polling based contention free access scheme, as specified in IEEE 802.15.6 standard. They also evaluated the performance in terms of network lifetime for both channel access schemes.

An amendment to the IEEE 802.15.6 standard has been introduced in [12] using the new Contention Priority (CP) dynamism which is based on the device’s queue length. This technique is contributed significantly to achieve optimized throughput, delay and data packet dropping rate.

In [13] authors have proposed an analytical model for the CSMA/CA based MAC layer of IEEE 802.15.6 which includes a probabilistic model and Markov chain for all user UPs. In consideration of saturated traffic environment they investigate the outcomes of deploying both EAP1 and RAP1 in terms of normalized network throughput and medium utilization of all UPs.

To cope with different UPs in a heterogeneous environment, authors in [14] have deployed a three dimensional Markov chain for modeling the backoff procedure of IEEE 802.5.6 CSMA/CA. An M/G/1/K queuing system for describing the packet queues in buffer in unsaturated conditions is also employed. The performance has been examined in terms of throughput and delay of all UPs.

A statistical method is proposed in [15] to estimate the probability of successful transmission in saturation, non saturation and time saturation model. Authors define time saturation model as a system model. In this model the system performance is varied following the operating time. They also investigated that the proposed method is performed equally well as the Markov chain method in case of both saturated and non saturated models. However, the proposed statistical method outperformed the Markov chain method in a time saturated model.

In [16] authors developed an analytical model for the calculation of performances metrics, i.e., normalized throughput, energy efficiency and mean frame service time in a non saturated environment. For heterogeneous traffic conditions a Markov chain model is employed which includes different access phases and different UPs as specified in standard. The research work concluded that EAP is not necessary because it degrades the system performance in terms of energy per bit, delay and system throughput.

In [17], the authors conclude that choice of access phase lengths play an important role in optimizing the WBAN performance. Moreover, they also found that deployment of EAP is not necessary in a WBAN; as short EAP with RAP brings about ineffective utilization of available bandwidth. Furthermore, their research work claims that it is not necessary to use all eight available user priorities to sufficiently achieve optimal performance of WBAN.

An 802.15.6 CSMA/CA MAC based Hybrid Lifetime Extended Directional Approach (LEDA) is proposed in [18]. This approach takes the benefit of directional superiority in order to save energy which uses multi beam directional mode in CSMA/CA and single beam directional mode in TDMA for transmission according to traffic varieties.

In this paper, we proposed hybrid node prioritization scheme to enhance the network lifetime of IEEE 802.15.6 standard in beacon enabled mode with superframe boundaries. Furthermore, mathematical expressions are derived for mathematical analysis of average delay, average throughput, average bandwidth efficiency, average energy consumption and network life time. This work is a significant contribution towards analysis and comparison of node prioritization schemes for IEEE 802.15.6 CSMA/CA standard. To validate our analytical results, we perform simulations in OMNET++/Mixim with different human postures.

The rest of the paper is organized as follows: WBAN architecture is discussed in Section 2, CSMA/CA procedure of IEEE 802.15.6 standard is described in Section 3. In Section 4, a detailed overview of node prioritization schemes for IEEE 802.15.6 standard is provided. Theoretical analysis of performance parameters is performed in Section 5, simulation results are discussed in Section 6, finally Section 7 concludes our research work.

## 2. WBAN Architecture

**Figure 1:**
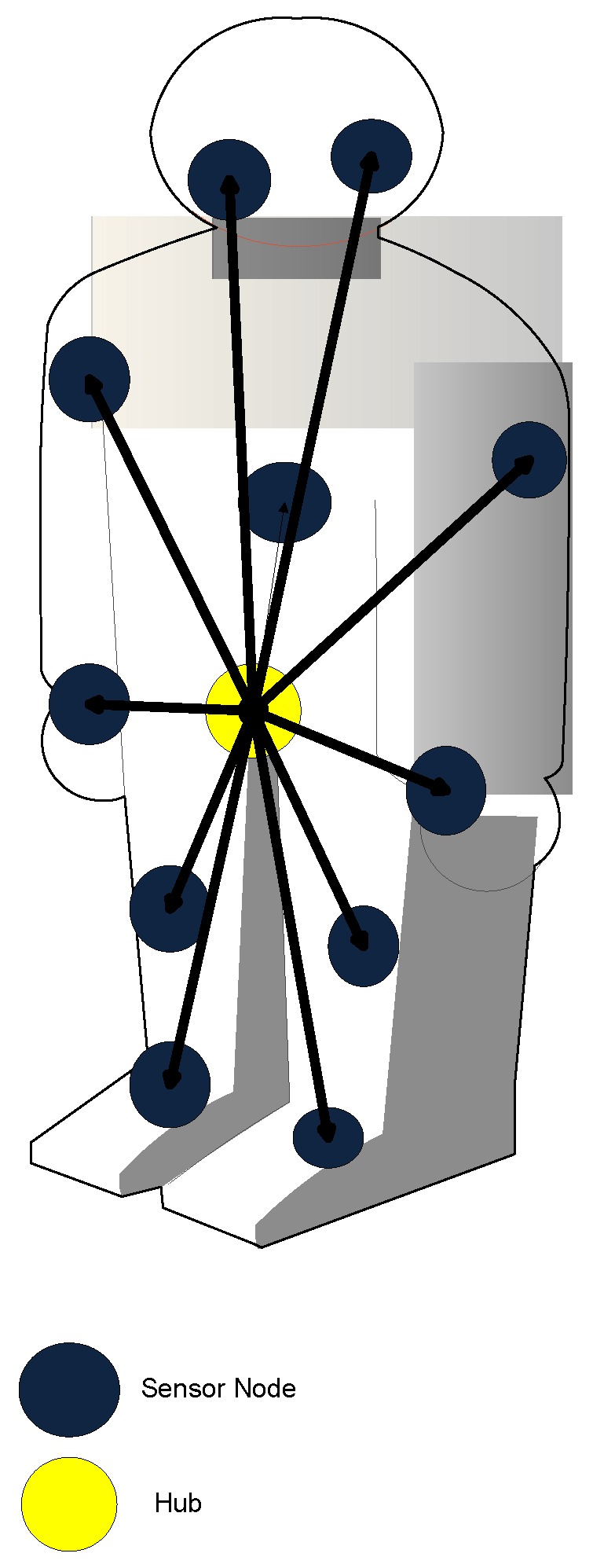
WBAN architecture.

**Figure 2:**
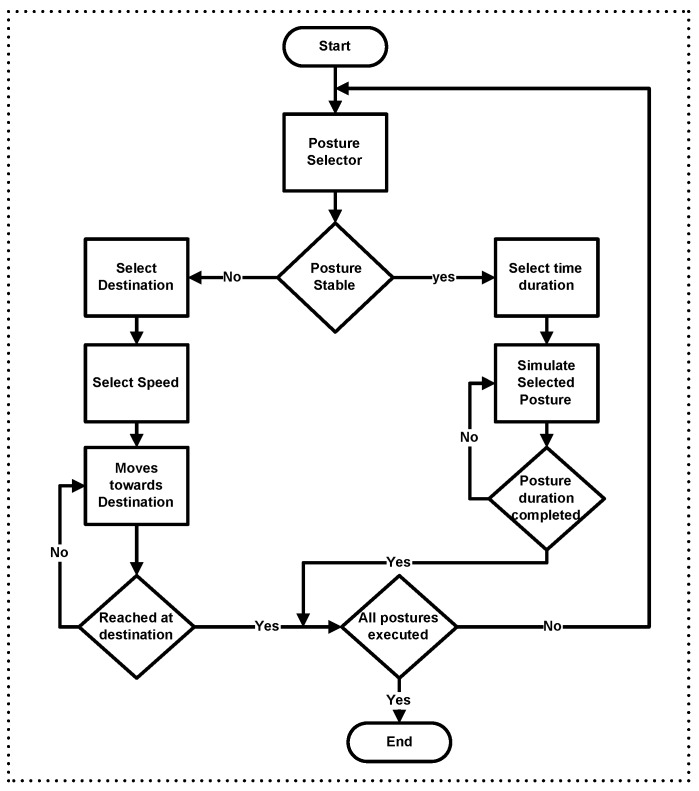
MOBAN structural Process.

**Figure 3:**
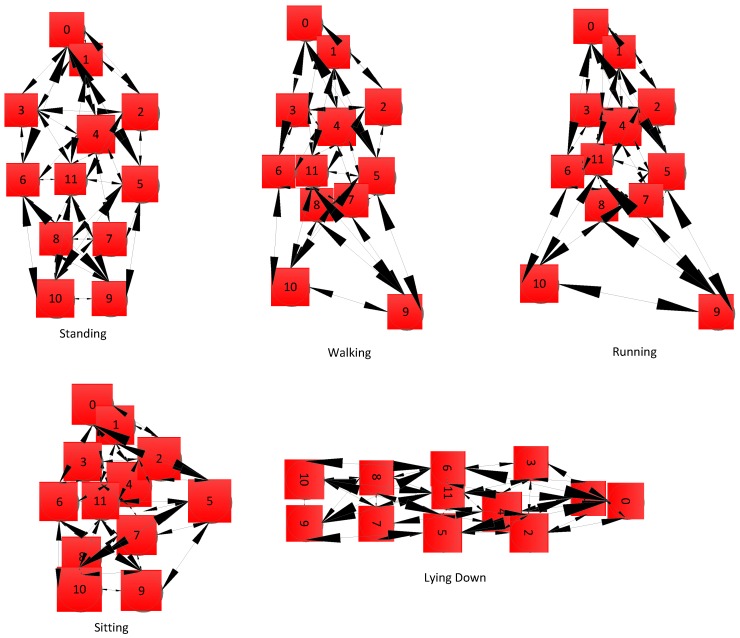
Posture orientations in OMNET++.

A WBAN is set up of several postures of the individual human body. The sensors associated with legs, arms and feet are mobile with in a limited range. In such cases, unstable signal integrity may temporally and spatially cause network performance degradation. To validate analytical results beyond real words scenario, we used MOBAN [19] mobility model for different human posture orientations. We used twelve sensor nodes in our simulation which are placed on human body as shown in Figure 1. The structural process of MOBAN is discussed in Figure 2. The posture orientations of human body in OMNET++ is shown in Figure 3.

## 3. IEEE 802.15.6 Standard

The IEEE 802.15.6 standard supports three physical layers, i.e., Narrow band (NB) PHY, Human Body Communication (HBC) PHY and Ultra Wide Band (UWB) PHY. NB PHY merely supports data transmission and reception in single frequency band. HBC PHY communicates in two frequency bands with bandwidth of 4 MHZ and centered at frequency of 16 and 27 MHZ respectively. UWB PHY operates in two frequency bands, these are high and low frequency bands. Each band is further divided into numerous channels. Each channel deals with a bandwidth of 499.2 MHZ. At MAC layer IEEE.802.15.6 specifies the channel access in one of the following modes. These are

1. Beacon mode with beacon period superframe boundaries
2. Non beacon mode with superframe boundaries
3. Non beacon mode without superframe boundaries

**Figure 4:**
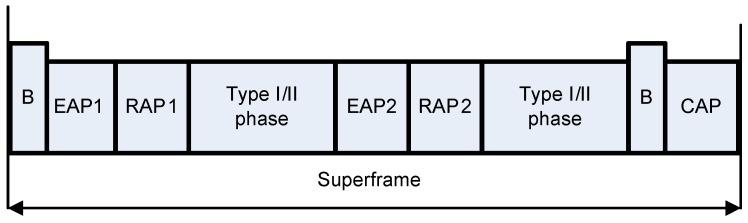
Superframe structure of beacon enabled mode.

A detailed overview of these channel access modes is described in [7]. We use CSMA/CA random channel access protocol in mode 1. In this mode, the hub is responsible for transmission of beacons in each beacon period. The superframe structure of this mode is shown in Figure 4.

In this mode, superframe is divided into Random Access Phase 1/2 (RAP 1/2), Exclusive Excess Phase 1/2 (EAP 1/2) and Contention Access Phase (CAP). IEEE 802.15.6 CSMA/CA standard allows to adjust the length of all access phases to zero except RAP.

**Figure 5:**
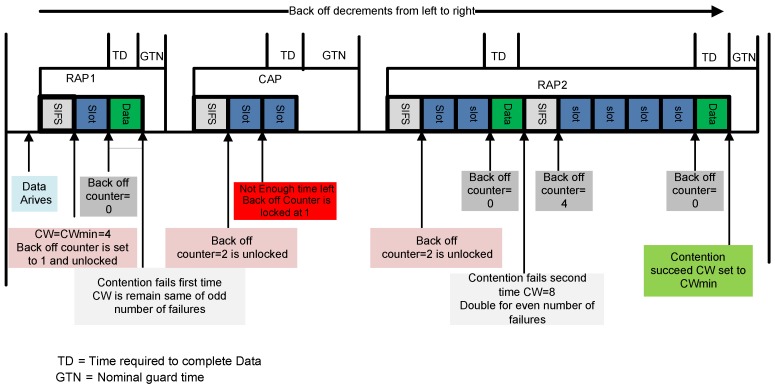
CSMA/CA procedure in IEEE 802.15.6.

EAP 1/2 are used to handle emergency traffic, while RAP 1/2 and CAP deal with normal traffic. In RAP 1, a node adjusted its backoff counter to a uniformly distributed random integer number over the interval [1, CW]. In idle channel conditions, a node decrements its backoff counter by one for each idle CSMA slots. When the backoff counter approaches zero, the data is transmitted. If the channel is occupied due to the frame transmission, a node locks its backoff counter and waits for idle channel for its frame transmission. The CW size remains unchanged for even number of frame retransmission failures. In case of odd number of retransmission failure, a node doubles its CW size and tries to access the channel again. For a successful frame transmission in RAP transmitting node resets its CW size to initial CW. The CSMA/CA procedure of IEEE 802.15.6 standard is portrayed in Figure 5.

## 4. Node Prioritization Schemes

The entire WBAN network is broken up into three types of sensors for critical, moderately critical and normal traffic. Critical nodes are assigned with Highest Priority (HP), moderately critical nodes have Moderate Priority (MP) and the nodes dealing with normal traffic are recognized as Low Priority (LP) nodes. Without the consideration of RTS/CTS handshake three node prioritization schemes for RAP are described below.

### 4.1. CW Assisted Traffic Prioritization

In this method, priority is assigned to critical, moderately critical and normal nodes with different sizes of CW. Nodes with minimum CW size achieve uppermost priority and highest CW size is assigned to lowest priority nodes. Priority assignment details of different nodes based on CW size for IEEE 802.15.6 CSMA/CA standard are given in Table 1.

We allocate three priorities $UP_{5}$, $UP_{3}$ and $UP_{1}$ to HP, MP and LP nodes. The CW size is increased from HP to LP nodes with following mathematical relations. $CW_{HP}\in \left(C_{min},C_{max}\right)$ is assigned to $UP_{5}$ nodes according to IEEE 802.15.6 standard. For MP nodes of $UP_{3}$, CW size is increased as $CW_{MP}\in \left[\left(CW_{min-HP}+2^{\delta }\right),2\left(CW_{min-HP}+2^{\delta }\right)\right]$ and CW size of LP nodes is further increased w.r.t MP nodes with following mathematical relation, $CW_{LP}\in \left[\left(CW_{MP-min}+2^{\delta +1}\right),2\left(CW_{MP-min}+2^{\delta +1}\right)\right]$. Here, *δ* is constant, its value is 2. IEEE 802.15.6 standard supports user priority from 0 to 7. In other words, this standard is capable of assigning users priorities. Smaller CW gets HP due to less average backoff time to access the channel. The allocation of different user priorities based on CW size allow beyond collision free channel access among different nodes in a network. However, without RTS/CTS handshake nodes associated with particular user priority group have a probability of collision due to same CW size.

**Figure 6:**
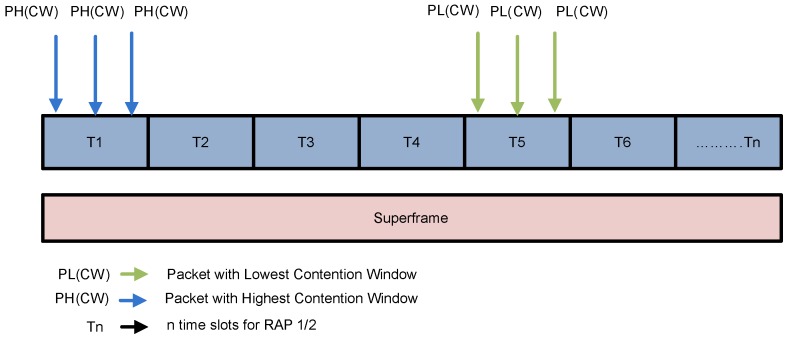
Packet collision scenario in CW assisted CSMA/CA.

The packet collision scenario with the assumption of two states S1 and S2 is shown in Figure 6.

- S1: Simultaneous arrival of three HP packets with PH(CW) arrived at T1.
- S2: Simultaneous arrival of three LP packets with PL(CW) arrived at T5.

**Figure 7:**
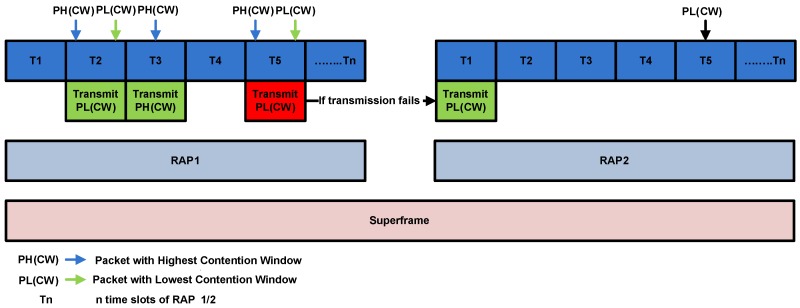
Collision avoidance scenario in CW assisted CSMA/CA.

The channel access mechanism without collision with the assumption of three states S1, S2 and S3 is illustrated in Figure 7.

- S1: Simultaneous arrival of one low priority packet and one high priority packet in T2.
- S2: Only high priority packet is arrived at T3.
- S3: Simultaneous Transmission of one high and one low priority packet at T5.

In S1 two packets: i.e., PH(CW) and PL(CW) arrived at same time. Due to the less average backoff time packet with PL(CW) accesses the channel without collision and becomes candidate for frame transmission. While, in S2 one packet PH(CW) arrives at a time. It successfully accesses the channel in absence of any competitor. In S3 both PL(CW) and PH(CW) simultaneously arrived at same time. However, PL(CW) accesses the channel successfully due to highest priority.

### 4.2. Transmission Power Assisted Traffic Prioritization

In this technique, the nodes of UP5, UP3 and UP1 are assigned priorities with three power levels; i.e., $P_{TX}$, $\alpha P_{TX}$ and $\beta P_{TX}$ to normal, moderately critical and critical traffic respectively, where $P_{TX}$<$\alpha P_{TX}$<$\beta P_{TX}$. In this scheme, we consider two scenarios of channel access.

**Figure 8:**
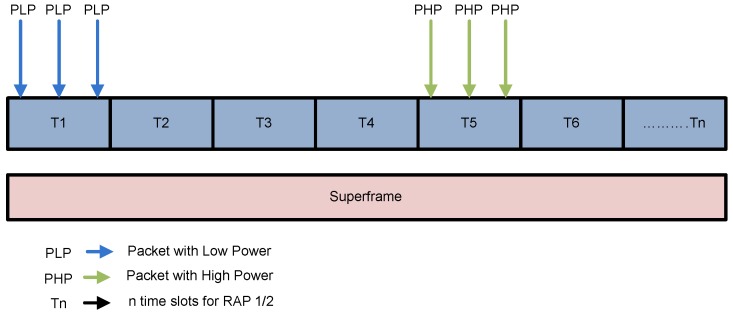
Packet collision scanario in Power assisted CSMA/CA.

In our scenario 1, two states are defined as S1 and S2 as shown in Figure 8.

- S1: Simultaneous arrival of more than one low power packets at T1.
- S2: simultaneous arrival of more than one high power packets at T5.

In these states, because of the packets of similar priority arrived at same time, hence they contribute to generate collision among them.

**Figure 9:**
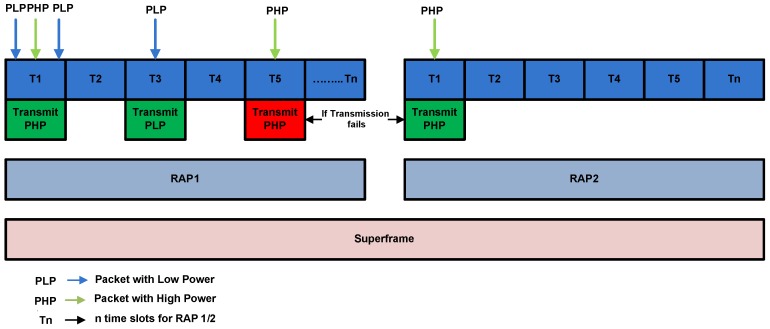
Packet collision avoidance scenario in power assisted CSMA/CA.

On the other hand, in scenario 2, we assume three states S1, S2 and S3 as portray in Figure 9.

- S1: Simultaneous arrival of one high power packet and one low power packet at T1.
- S2: one low power packet arrived at T3.
- S3: one high power packet arrived at T5.

When the packets of different priorities arrived at the same time, they are received at destination without collision. In S1 one packet with transmission power $\beta P_{TX}$ and two packets with transmission power $P_{TX}$ arrived at the same time, as per prioritization policy, the packet with transmission power $\beta P_{TX}$ successfully accesses the channel without any collision. In RAP 1, If transmission failure of a node occurs and there is not enough time for packet retransmission, it retransmits packet in RAP 2. The details of CW size, transmission power and other attributes for both prioritized and normal networks are given in Table 2.

### 4.3. Hybrid Node Prioritization

In IEEE 802.15.6 standard nodes are divided into different UPs. All nodes in a UP have same CW sizes. Hence, chance of collision amongst the nodes of same UP can not be neglected. In this scheme, we further prioritized nodes in each UP by assigning different transmission power levels i.e., $P_{tx}$, $\alpha P_{tx}$, $\beta P_{tx}$, $\lambda P_{tx}$, where, α<β<λ. In hybrid node prioritization scheme the nodes belonging to different UPs are prioritized on the basis of different CW sizes at MAC layer and the nodes belong to each UP are further prioritized based on transmission power level at physical layer. The working mechanism of our proposed scheme is as follows.

Initially, at physical layer, our proposed scheme calculates path loss based on Received Signal Strength Indicator (RSSI) through beacon packet transmission from the sink node. Using path loss, a node decides on the basis of threshold whether or not to participate in contention for channel access. RSSI is function of transmitted power and distance. Moreover, path loss fluctuates with posture variations. Additionally, a node with higher transmission power has a better opportunity to participate in contention for channel access at MAC layer. The packets with transmission power lower than threshold are discarded in any competition for channel access. This scheme reduces the number of participants for channel access. Therefore, it also reduces the possibility of collision amongst the nodes. Later, at MAC layer, the channel access priorities are allocated based on CW size. Therefore, a node with smaller CW size has lesser average backoff time to access the channel.

The benefits of hybrid prioritization scheme are two-fold.

- This scheme avoids the number of retransmissions by means of making a decision on the basis of RSSI. When an odd number of retransmission failure occurs, IEEE 802.15.6 standard doubles its backoff counter. In this regard, it reduces the average backoff time to access the channel.
- Furthermore, it minimizes the number of participants in contention of channel access. Hence, the probability of collision amongst the nodes is diminished.

For a special scenario, when more than one node access the channel at same time then the proposed scheme takes the decision on the basis of following rules.

- Primarily, our proposed scheme decides on the basis of the node IDs, whether it belongs to same UP. Hence, a node belonging to the highest UP is preferred for packet transmission.
- In contrast, for the same UP, the decision is be made on the basis of transmission power levels.

**Figure 10:**
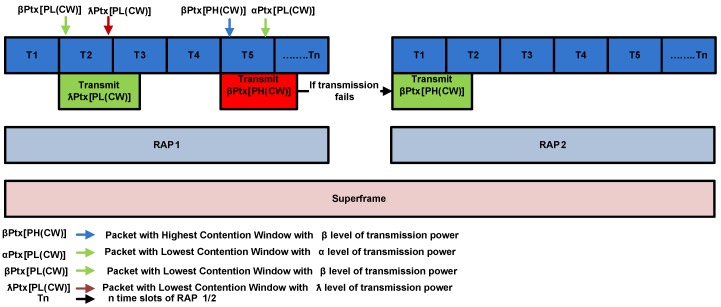
Hybrid node prioritization.

Although, it is possible that the transmission power level of lower priority packet is higher than higher priority packet. However, the priority is assigned on the basis of node ID. An example scenario for channel access mechanism of hybrid node prioritization scheme with the assumption of two states S1 and S2 is discussed in Figure 10.

- S1: Simultaneous arrival of two high priority packets with *λ* and *β* transmission power levels respectively at T2.
- S2: Simultaneous arrival of one low priority packet with *β* transmission power level and one high priority packet with *α* power level.

This node prioritization scheme ensures the CW size of a node in any time slot. A node having lesser CW size accesses the channel earliest. In case of S1, $\lambda P_{tx}\left[PL\left(CW\right)\right]$ is transmitted because of high amount of transmission power. As, CW size of both sensor nodes is same. $\alpha P_{tx}\left[PL\left(CW\right)\right]$ is transmitted in S2, as it belongs to nodes of higher UP. As a result, this technique contributes to mitigate the collision amongst the nodes of the same UP.

## 5. Theoretical Analysis

We derive the mathematical expression to calculate our performance measures: i.e., average delay, throughput, bandwidth efficiency and energy consumption for both prioritized and normal networks. Notations used for theoretical analysis are listed in Table 3. Before mathematical calculations, we consider following assumptions.

1. Packet error rate due to noise is ignored.
2. In each prioritized set of nodes the collision probability of every node is constant .
3. Each node in the network always has a packet to send. (Saturated Regime)
4. Losses due to buffer overflow are ignored.

### 5.1. Delay, Throughput and Bandwidth Efficiency Calculations

#### 5.1.1. CW Assisted Prioritization

In case of priority with CW size, we calculate the delay for each prioritized node using the following mathematical expressions

(1) $$Delay_{HP}=T_{SB-HP}+T_{TX}+T_{ACK}+2T\rho SIFS+2\tau$$

(2) $$Delay_{MP}=T_{SB-MP}+T_{TX}+T_{ACK}+2T\rho SIFS+2\tau$$

(3) $$Delay_{LP}=T_{SB-LP}+T_{TX}+T_{ACK}+2T\rho SIFS+2\tau$$

For *n* number of maximum backoff periods, the probability that a sensor node can successfully access the channel is given by

(4) $$P_{s}=\sum_{i=1}^{n}p_{a}{\left(1-p_{a}\right)}^{\left(i-1\right)}$$

where $p_{a}$ is the probability that a node can access the ideal channel at the end of each backoff period. For *k* number of nodes in the network $p_{a}$ is given by

(5) $$P_{a}={\left(1-q\right)}^{\left(k-1\right)}$$

where *q* is the probability that a network device is transmitting at any time. The average number of backoff periods, *R* is calculated in [20] as follows

(6) $$R=\left(1-p_{s}\right)n+\sum_{i=1}^{n}p_{a}{\left(1-p_{a}\right)}^{\left(i-1\right)}$$

Average back of time ($T_{SB}$) for successfully transmitted packet for each prioritized node in UP5, UP3 and UP1 is calculated as

(7) $$T_{SB-HP}=\frac{CW_{min}\times T_{slot}}{2}\times R$$

(8) $$T_{SB-MP}=\frac{\left(CW_{min}+2^{\delta }\right)\times T_{slot}}{2}\times R$$

(9) $$T_{SB-LP}=\frac{\left(CW_{min}+2^{\delta }+2^{\delta +1}\right)\times T_{slot}}{2}\times R$$

where $T_{TX}$, $T_{I-ACK}$ and $T_{B-ACK}$ is calculated in [10] as:

$$T_{TX}=T_{\rho }+T_{phy}+\frac{MHR+X+FTR}{R_{data}}$$

$$T_{I-ACK}=T_{\rho }+T_{phy}+\frac{MHR+FTR}{R_{data}}$$

$$T_{B-ACK}=T_{\rho }+T_{phy}+\frac{MHR+X^{{}^{\prime }}+FTR}{R_{data}}$$

In case of unsuccessful transmission the $T_{SB}$ is denoted by $T_{DB}$ and calculated for each prioritized node with following mathematical expressions

(10) $$T_{DB-HP}=\frac{CW_{min}\times T_{slot}}{2}\times \left(R+1\right)$$

(11) $$T_{DB-MP}=\frac{\left(CW_{min}+2^{\delta }\right)\times T_{slot}}{2}\times \left(R+1\right)$$

(12) $$T_{DB-LP}=\frac{\left(CW_{min}+2^{\delta }+2^{\delta +1}\right)\times T_{slot}}{2}\times \left(R+1\right)$$

Average delay of unprioritized network is given by

(13) $$Delay_{UN}=\frac{1}{N}\sum_{i=1}^{n}Delay\left(i\right)$$

Average delay of prioritized network is obtained as

(14) $$Delay_{PN}=\frac{1}{N_{HP}}\sum_{i=1}^{n}Delay\left(i\right)+\frac{1}{N_{MP}}\sum_{j=1}^{n}Delay\left(j\right)+\frac{1}{N_{LP}}\sum_{k=1}^{n}Delay\left(k\right)$$

Throughput is defined as the ratio of payload size (*X*), to the total transmission delay.

In case of unprioritized network Maximum Throughput (*MT*) is calculated as

(15) $$MT_{UN}=\frac{X}{Delay_{UN}}$$

For prioritized network it is given by

(16) $$MT_{PN}=\frac{X}{Delay_{PN}}$$

Bandwidth efficiency (ρ) is used to determine the spectral utilization of 1EEE 802.15.6 CSMA/CA. It is the ratio of maximum throughput to the data rate.

For unprioritized network it is given by

(17) $$\rho_{UN}=\frac{MT_{UN}}{R_{Data}}$$

In case of prioritized network it is given by

(18) $$\rho_{PN}=\frac{MT_{PN}}{R_{Data}}$$

#### 5.1.2. Power Assisted Prioritization

In the case of power assisted prioritization, the CW size for each node remains same. The delay, throughput and bandwidth efficiency depend on the number of collisions and probability of frame rejection, which depends on the number of allowed packet retransmissions (*m*). For the transmission of first packet the probability of packet transmission failure is $\left(1-P_{S}\right)$, where $P_{S}$ is probability of successful packet transmission in particular time slot. The probability of packet transmission failure is ${\left(1-P_{S}\right)}^{m+1}$. Hence, $P_{S}$ is greater for a set of nodes with power $\beta P_{TX}$.

#### 5.1.3. Hybrid Prioritization

The hybrid prioritization scheme takes advantage of both prioritization schemes. Hence, average backoff time for channel access is reduced and it also avoids packet collisions due to prioritization based on transmission power levels to each node in same user priority. As a result, it increases the throughput and bandwidth efficiency and reduces the delay of network.

### 5.2. Energy Consumption Calculations

#### 5.2.1. Power Assisted Prioritization

In case of power assisted prioritization, we assigned the transmission powers $P_{tx}$, $\alpha P_{tx}$ and $\beta P_{tx}$ to the nodes of LP, MP and HP respectively.

In case of unsuccessful transmission, the energy consumption of each prioritized node is obtained as

(19) $$E_{DB}=P_{Idle}\times T_{DB}$$

(20) $$E_{DC-LP}=\left(R+1\right)\times \left[P_{RX-TX}\times t_{RX-TX}+T_{C}\times P_{TX}+P_{TX-RX}\times t_{TX-RX}\right]$$

(21) $$E_{DC-MP}=\left(R+1\right)\times \left[P_{RX-TX}\times t_{RX-TX}+T_{C}\times \alpha P_{TX}+P_{TX-RX}\times t_{TX-RX}\right]$$

(22) $$E_{DC-HP}=\left(R+1\right)\times \left[P_{RX-TX}\times t_{RX-TX}+T_{C}\times \beta P_{TX}+P_{TX-RX}\times t_{TX-RX}\right]$$

(23) $$E_{D-LP}=E_{DB}+E_{DC-LP}$$

(24) $$E_{D-MP}=E_{DB}+E_{DC-MP}$$

(25) $$E_{D-HP}=E_{DB}+E_{DC-HP}$$

In case of successful transmission the energy consumption for each prioritized node is calculated as

(26) $$E_{SB}=P_{Idle}\times T_{SB}$$

(27) $$E_{SC-LP}=N_{C}\times \left[P_{RX-TX}\times t_{RX-TX}+T_{C}\times P_{TX}+P_{TX-RX}\times T_{TX-RX}\right]$$

(28) $$E_{SC-MP}=N_{C}\times \left[P_{RX-TX}\times t_{RX-TX}+T_{C}\times \alpha P_{TX}+P_{TX-RX}\times T_{TX-RX}\right]$$

(29) $$E_{SC-HP}=N_{C}\times \left[P_{RX-TX}\times t_{RX-TX}+T_{C}\times \beta P_{TX}+P_{TX-RX}\times T_{TX-RX}\right]$$

(30) $$E_{TX-LP}=T_{TX}\times P_{TX}$$

(31) $$E_{TX-MP}=T_{TX}\times \alpha P_{TX}$$

(32) $$E_{TX-HP}=T_{TX}\times \beta P_{TX}$$

(33) $$E_{S-LP}=E_{TX}+E_{SC}+E_{SB}$$

(34) $$E_{S-MP}=E_{TX-MP}+E_{SC-MP}+E_{SB}$$

(35) $$E_{S-HP}=E_{TX-HP}+E_{SC-HP}+E_{SB}$$

Total energy consumption of a sensor node is given by

(36) $$E_{T}=E_{S}+E_{D}$$

The lifetime of sensor node is calculate as

(37) $$T_{node}=\frac{E_{B}}{E_{T}}\times T$$

Average life time of a node in unprioritized network contain *N* number of nodes is obtained as

(38) $$T_{UP_{node}}=\frac{1}{N}\sum_{k=0}^{n}T_{node}\left(k\right)$$

Average life time of a sensor node in prioritized network consists of *N* number of nodes is given by

(39) $$T_{PN_{node}}=\frac{1}{N_{LP}}\sum_{i=0}^{n}T_{node}\left(i\right)+\frac{1}{N_{MP}}\sum_{j=0}^{n}T_{node}\left(j\right)+\frac{1}{N_{HP}}\sum_{k=0}^{n}T_{node}\left(k\right)$$

#### 5.2.2. CW Assisted Prioritization

CW assisted prioritization requires extra amount of retransmissions w.r.t power assisted node prioritization. Ultimately, it is not energy efficient prioritization scheme.

#### 5.2.3. Hybrid Prioritization

In case of hybrid prioritization the energy consumption in packet collisions and retransmissions is reduced.

## 6. Results and Discussions

In this section, we present simulation and numerical analytical results of hybrid node prioritization performance evaluation in terms of delay, throughput, bandwidth efficiency, energy consumption and network lifetime for different scenarios. We neglected the electrical characteristics of human body and considered the on body propagation effects only. The values of these performance parameters are calculated from equations delivered in this paper. We assume WBAN is formed in star topology with *N* heterogenous nodes. The number of nodes in a network can be determined as $N=\sum_{i=0}^{7}s_{i}$, where $s_{i}$ is the number of nodes in each class. We have made a comparison of hybrid node prioritization with CW assisted node prioritization, Power assisted node prioritization and unpriortized network on OMNET++/MIXIM simulation framework. The OMNET++ is a scalable simulation framework based on discrete event simulation technology. Simulation results are conducted under ideal transmission conditions. We use standing posture for stable network and running and walking postures to support mobility in WBAN. The relevant simulation parameters and their values are listed in Table 4. Energy consumption data for nodes are derived from CC2440 chip [21].

### 6.1. Effect of Payload Size

**Figure 11:**
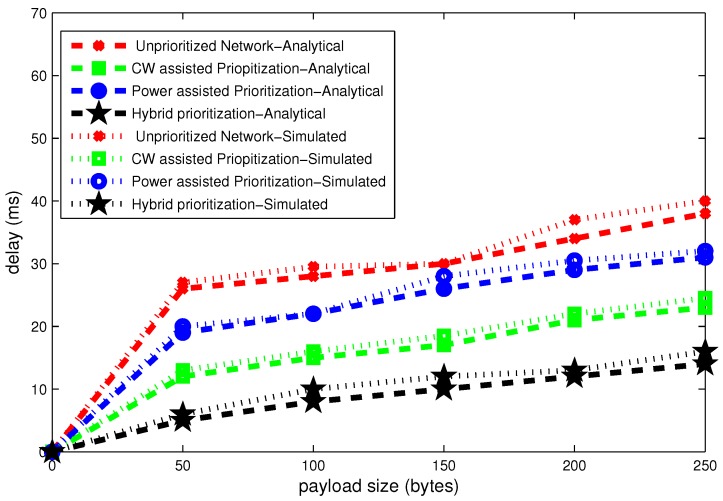
Effect of payload size on delay.

**Figure 12:**
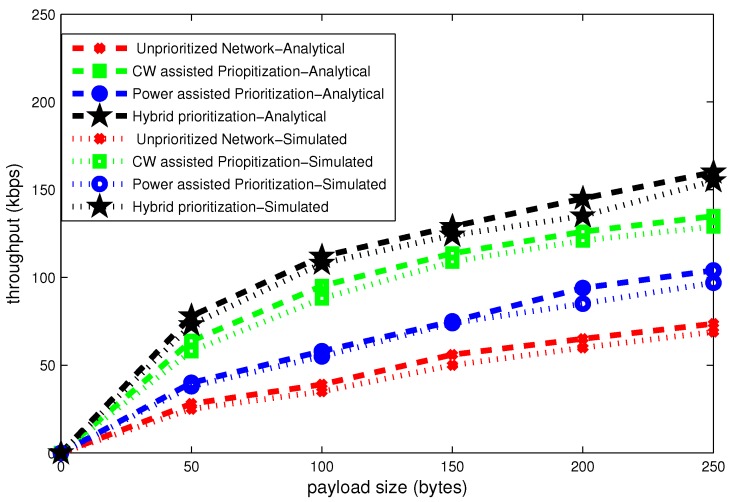
Effect of payload size on throughput.

**Figure 13:**
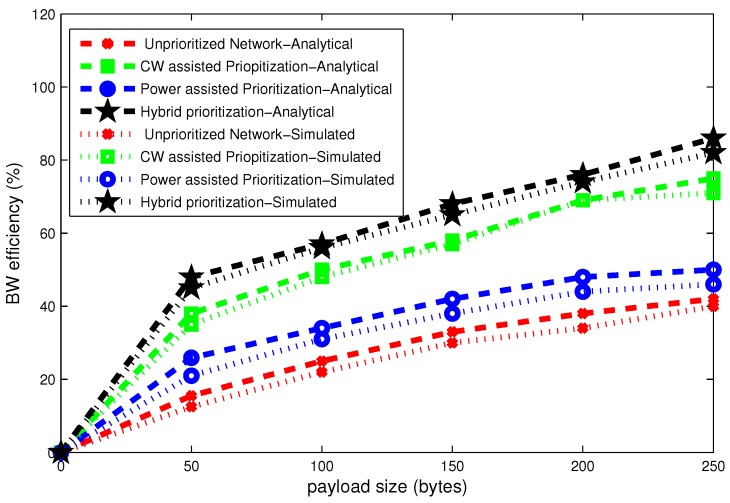
Effect of payload size on bandwidth efficiency.

The average delay results for stable network computed according to Equations (13) and (14) are shown in Figure 11, Figure 12 and Figure 13. These figures present delay, throughput and band width efficiency as a function of payload size.

In Figure 11, the performance of upriortized network and power assisted node prioritization is worse than hybrid and CW assisted node prioritization schemes in terms of average delay. It is clear that all twelve nodes have the same CW size in an unprioritized network. Hence, numerous collision occurs and all nodes go to backoff on several occasions. In the case of CW assisted node prioritization, the possibility of collision amongst the nodes belong to same UP is not neglected. Hybrid node prioritization scheme performs better than CW assisted prioritization because it avoids collisions in each UP. Probability of successful transmission in case of hybrid prioritization is higher due to high amount of transmission power. The unnecessary packet retransmissions increase the network delay. IEEE.802.15.6 CSMA/CA also doubles the CW size in case of even number of retransmission failures. As a result, average backoff time also increases. It is concluded that number of backoffs and retransmission failures are important factors which influence the delay.

It can be clearly seen in Figure 12, that the throughput grows as a function of payload size. Average throughput for stable network computed according to Equations (15) and (16). It is clearly seen that minimum delay scheme in Figure 11 experiences with maximum throughput. Analytical and simulation results are approximately match each other.

Bandwidth efficiency is directly proportional to the throughput. It is calculated according to Equations (17) and (18) for same attributes used for delay and throughput analysis. The scheme which deals with maximum throughput in Figure 12 achieves maximum bandwidth efficiency in Figure 13.

### 6.2. Effect of Data Rate

**Figure 14:**
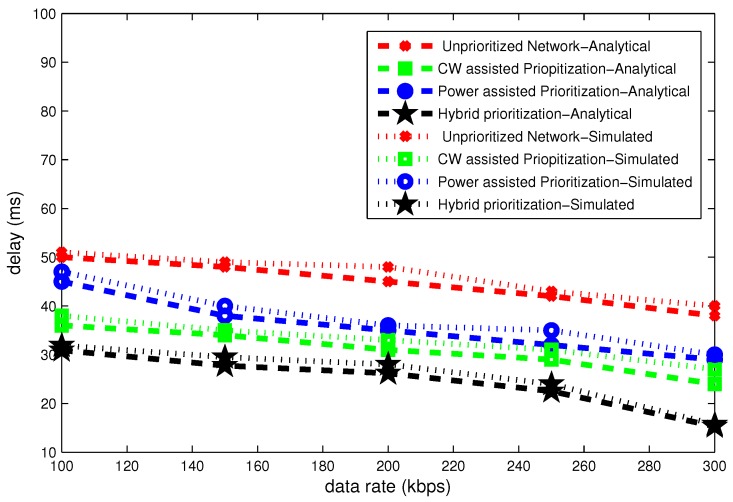
Effect of data rate on delay.

**Figure 15:**
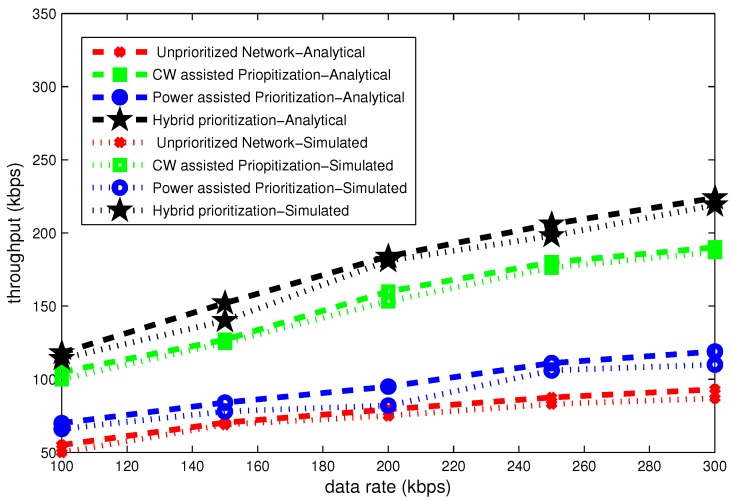
Effect of data rate on throughput.

**Figure 16:**
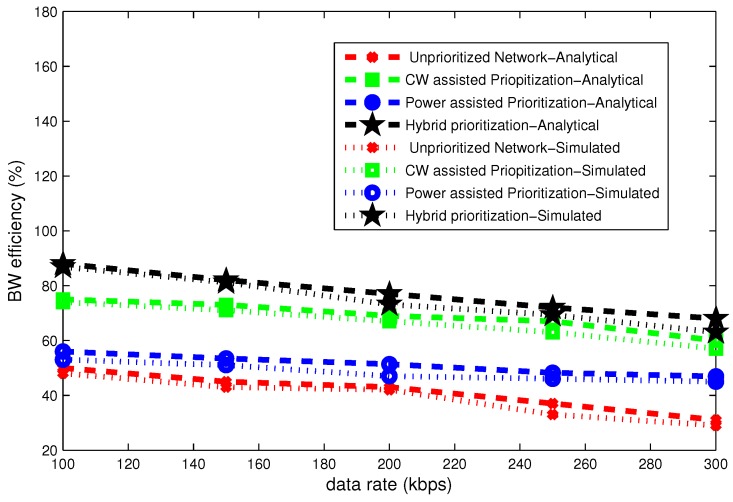
Effect of data rate on bandwidth efficiency.

The Figure 14, Figure 15 and Figure 16 present delay, throughput and band width efficiency as a function of data rate. The delay declines as a function of data rate and throughput grows with data rate. In case of unpriortized network, a lot of collision occurs and all nodes go to backoff several times. Hybrid node prioritization scheme once again outperforms all node prioritization schemes by the virtue of collision avoidance in each UP. Average number of backoffs are reduced in hybrid node prioritization scheme.

Figure 14 depicts that delay declines gradually as a function of data rate. At data rate of 100 kbps maximum delay of 50 ms occurs in case of unprioritized network. On the other hand, hybrid node prioritization attains minimum delay of 30 ms.

Figure 15 portrays the throughput for different data rates. It can be seen in this figure, the throughput steadily grows as a function of data rate. At data rate of 100 kbps, the throughput is highest for hybrid node prioritization and lowest in case of unprioritized network. Its value is 200 kbps and roughly 80 kbps. For the data rate of 300 kbps, the throughput of hybrid node prioritization approaches to 200 kbps. It value is approximately 70 kbps for unprioritized network. Simulation results also validate the analytical results.

Bandwidth efficiency is inversely proportional to data rate. Hybrid node prioritization is most bandwidth efficient amongst all as shown in Figure 16.

### 6.3. Influence of Mobility on Network Performance

We analyze the effect of posture variations on the performance of network in terms of delay, throughput and bandwidth efficiency. Postural mobility causes connection variations among different nodes of the human body. Relative positions of receiver with respect to transmitter on the body are important for path loss; however the relative position with respect to body posture, frequent variations in body movement, shadowing and fading are also key factors. We used jakes fading channel to analyze the impact of human body postures on the performance of WBAN.

#### 6.3.1. Effect of Payload Size

**Figure 17:**
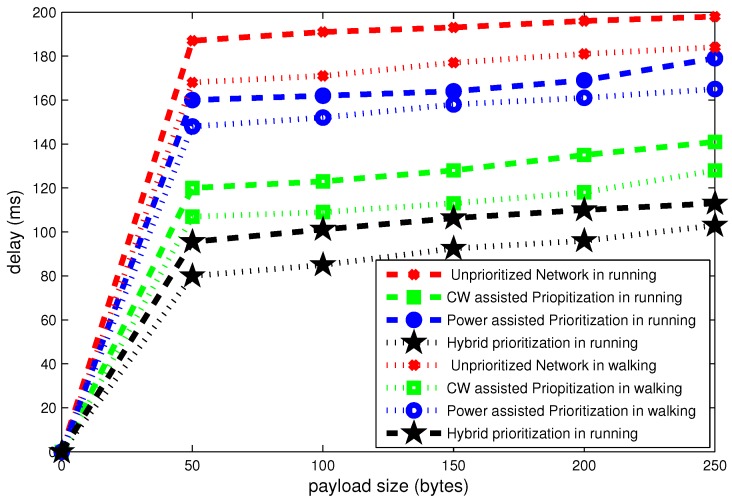
Effect of payload size on delay in mobile postures.

**Figure 18:**
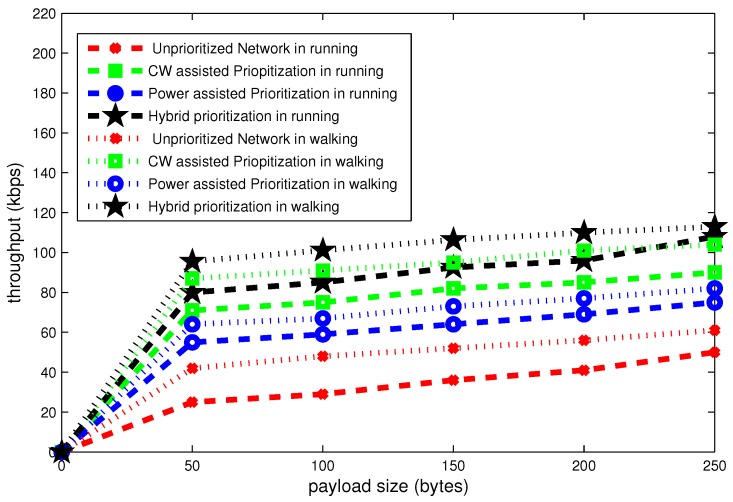
Effect of payload size on throughput in mobile postures.

**Figure 19:**
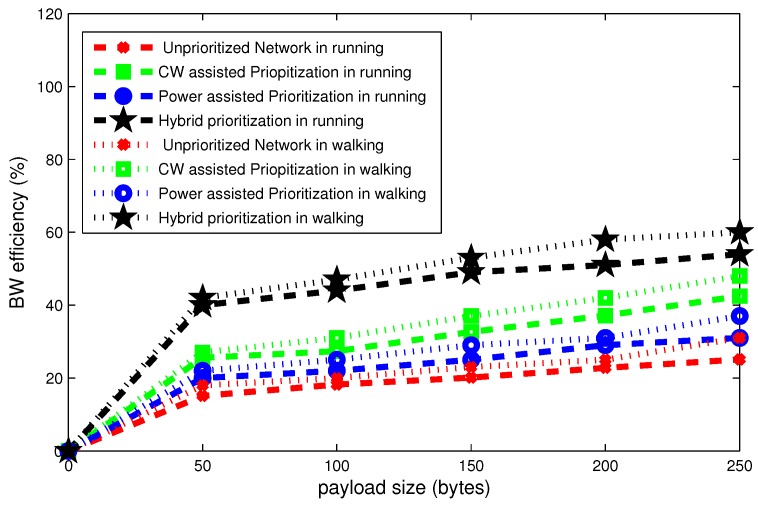
Effect of payload size on bandwidth efficiency in mobile postures.

It is observed in Figure 17, Figure 18 and Figure 19 that posture variations have significant effect on the overall performance of network.

Figure 17 shows the delay for different payload size. It can be seen in this figure that delay grows as a function of payload size. For the payload size of 50 bytes, the unprioritized network experiences the delay of around 190 ms in running condition and 165 ms in walking posture. The aggregate difference is about 25 ms due to posture variations. The hybrid node prioritization scheme attains minimum delay about 78 ms in walking condition. Total difference in delay due to postural changes from walking to running is 10 ms.

Figure 18 depicts that the throughput gradually declines as a function of payload size. For 50 bytes, hybrid node prioritization achieves maximum throughput of 98 kbps in walking position, which is an improvement of 10 kbps as compared to running position. Whereas, the value of throughput is minimum in case of unprioritized network. For this network, the throughput is 20 kbps and 40 kbps for running and walking postures, respectively, at 50 kbps.

Figure 19 shows that the bandwidth efficiency progressively raises for different payload sizes. It can be seen in the figure that the bandwidth efficiency grows as a function of payload size. For the payload size of 50 bytes, the unprioritized network attains 10% bandwidth efficiency; whereas, its value is 42% in the case of hybrid node prioritization. For walking conditions at the payload size of 250 bytes, the bandwidth efficacy is 60% and 25% for hybrid node prioritization and unprioritized network, respectively. Furthermore, there is 5% difference in bandwidth efficiency due to posture change at payload size of 250 bytes.

#### 6.3.2. Effect of Data Rate

**Figure 20:**
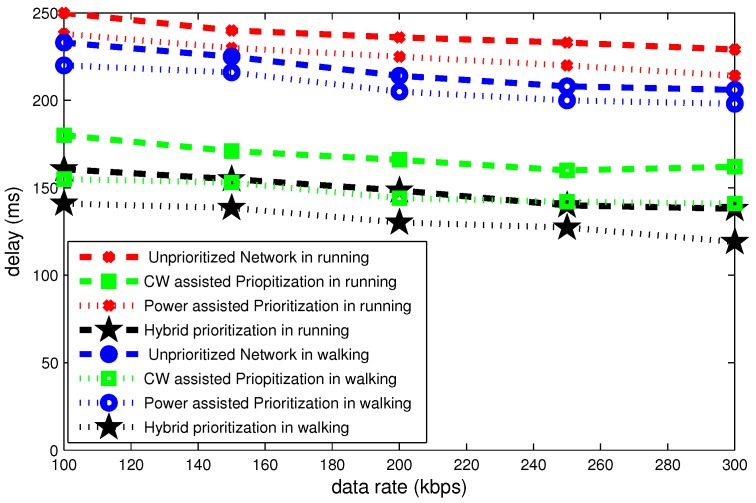
Effect of data rate on delay in mobile postures.

**Figure 21:**
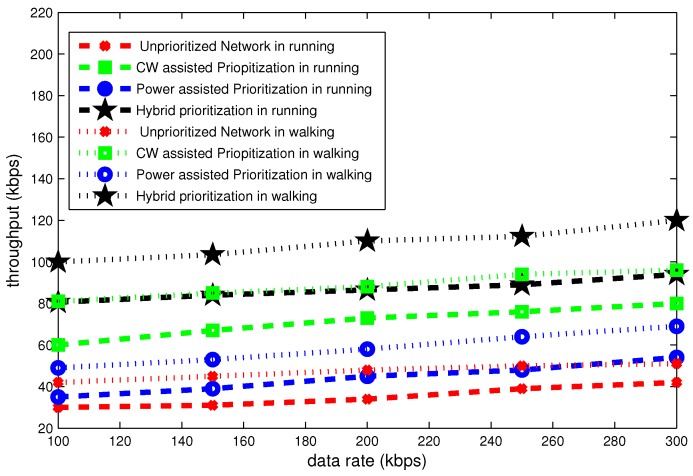
Effect of data rate on throughput in mobile postures.

**Figure 22:**
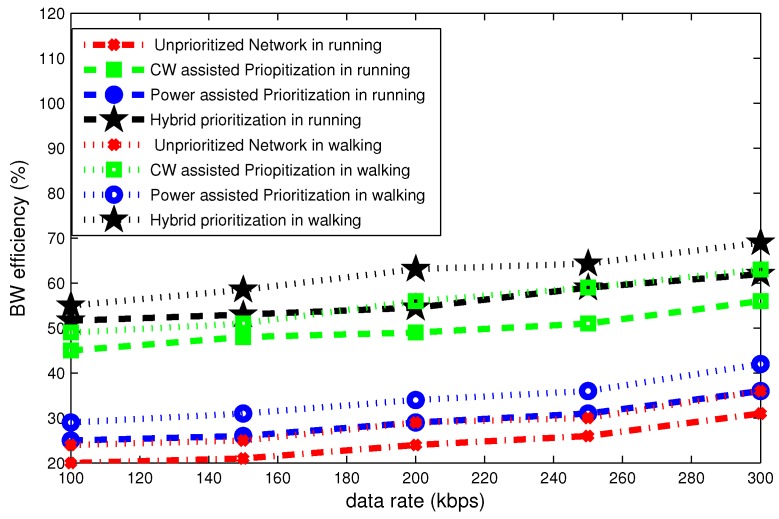
Effect of data rate on bandwidth efficiency in mobile postures.

Figure 20, Figure 21 and Figure 22 illustrate the delay, throughput and bandwidth efficiency in walking and running postures as a function of data rate.

Figure 20 depicts that delay declines gradually as a function of data rate. At data rate of 100 kbps maximum delay of 250 ms occurs in the case of an unprioritized network. On the other hand, hybrid node prioritization attains minimum delay of 160 ms in running condition. The total gap in delay is 20 ms in two aforementioned postures.

Figure 21 portrays the throughput for different data rates. It can be seen in this figure, the throughput steady grows as a function of data rate. At data rate of 100 kbps the throughput is highest for hybrid node prioritization and lowest in the case of an unprioritized network. Its value is 100 kbps and 30 kbps in standing posture. On average the total difference in throughput due to posture changes is approximately 20 kbps.

Figure 22 represents that bandwidth efficiency increases gradually as a function of data rate. The bandwidth efficiency curves are very important to observe the saturation tendency for different payload sizes and data rates. For the data rate of 300 kbps, the maximum bandwidth efficiency is roughly 70% in case of hybrid prioritization. Whereas, its minimum value is 25% in case of unprioritized network. The total gap in bandwidth efficiency due to posture variation is approximately 10%.

### 6.4. Energy Consumption

**Figure 23:**
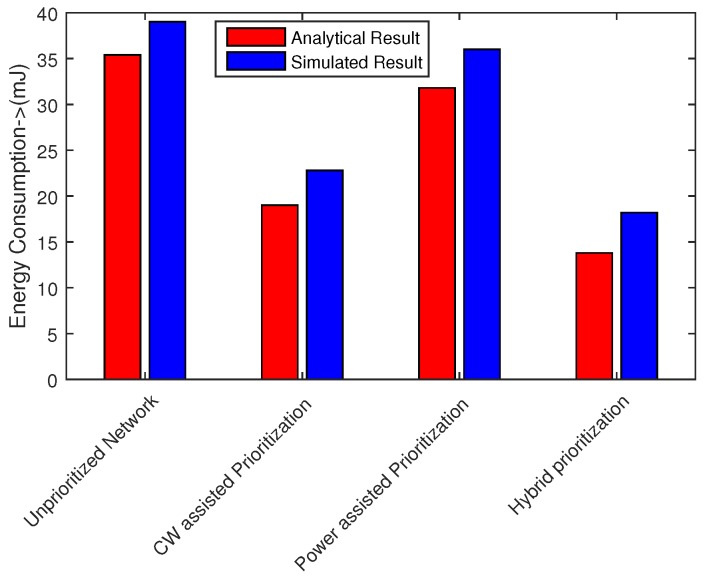
Average network energy consumption.

Total energy consumption of the network is the sum of transmission power, receiving power and power consumed in packet collisions. Transmission power has highest influence on energy consumption of network. Figure 23 shows the energy consumption analysis computed according to Equation (36). In our simulations, we used well-known CC2420 transceiver to derive typical power consumption data for sensor nodes. The results confirm that hybrid node prioritization scheme is effective in energy saving. This scheme avoids packet collisions and retransmissions due to prioritization based on CW size and transmission power level. Normal network experiences with worse performance in the absence of any prioritization mechanism. Our proposed scheme save 85% as compared to unpriortized network and consumes 25% less energy w.r.t IEEE 802.15.6 network. Analytical and simulation results also roughly match each other.

### 6.5. Network Lifetime

**Figure 24:**
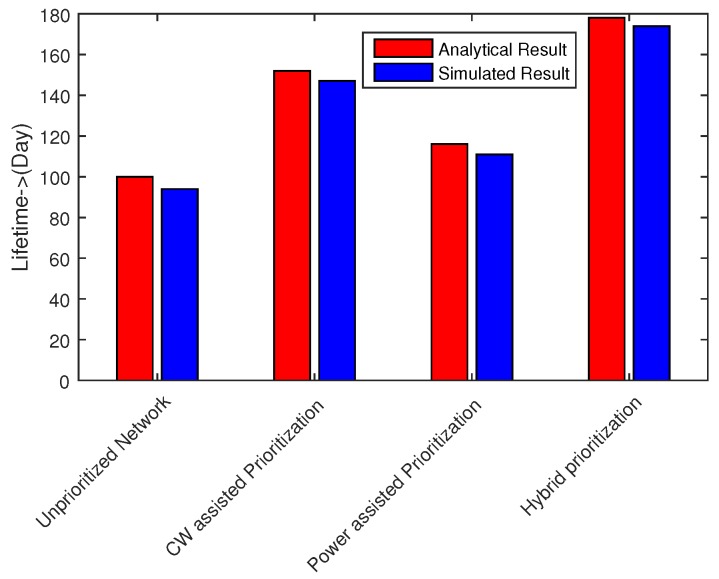
Network lifetime.

The lifetime of a sensor network is defined as the time duration from the Beginning of network operation to the instance when any sensor node runs out of its energy. We evaluate the lifetime of both stable and mobile network with the consideration that the twelve nodes are equipped with 560 mAH battery capacity. Considering Figure 24, it is apparent that hybrid node prioritization prolongs the network lifetime to close to 177 days, while the lifetime of CW assisted IEEE 802.15.6 CSMA/CA standard is approximately 150 days. Our proposed prioritization scheme for 802.15.6 CSMA/CA can significantly extend the network lifetime, which is appropriate for WBAN.

## 7. Conclusions and Future Work

Network life time extension in WBAN is the prime research issue for both academia and industry. This paper proposes a hybrid node prioritization scheme in IEEE 802.15.6 CSMA/CA standard to reduce the energy consumption and prolong network lifetime. Analytical and simulation results demonstrate that energy consumption of hybrid node prioritization is reduced to 25% as compared to CW assisted IEEE.802.15.6 CSMA/CA. The network lifetime is almost 77 days greater as compared to IEEE 802.15.6 CSMA/CA standard. Furthermore, our proposed scheme also outperforms all node prioritization schemes in terms of delay, throughput and bandwidth efficiency. One major problem with hybrid node prioritization is that it requires advanced nodes in terms of transmission power for each user priority group. Optimizing transmission power control of hybrid node prioritization to achieve optimum performance is our future work.

## Figures and Tables

**Table 1:** Contention window bounds for IEEE 802.15.6 CSMA/CA.

UP	Traffic Type	${\mathrm{CW}}_{\min}$	${\mathrm{CW}}_{\max}$
0	Background	16	64
1	Best effort	16	32
2	Excellent	8	32
3	Video	8	16
4	Voice	4	16
5	Medical data	4	8
6	High priority medical data	2	8
7	Emergency Reports	1	4

**Table 2:** Simulation parameters for prioritized and normal network.

**Hybrid Node Prioritization**			
**UP**	**No. of Nodes**	$\mathrm{CW}\in (C_{\min},C_{\max})$	**Transmission Power**
$UP_{5}$	4	(4,8)	$P_{tx}$, $\alpha P_{tx}$, $\beta P_{tx}$, $\lambda P_{tx}$
$UP_{3}$	4	(8,16)	$P_{tx}$, $\alpha P_{tx}$, $\beta P_{tx}$, $\lambda P_{tx}$
$UP_{1}$	4	(16,32)	$P_{tx}$, $\alpha P_{tx}$, $\beta P_{tx}$, $\lambda P_{tx}$
**Power Assisted Node Prioritization**			
**UP**	**No. of Nodes**	$\mathrm{CW}\in (C_{\min},C_{\max})$	**Transmission Power**
$UP_{5}$	4	(16,32)	$\beta P_{tx}$
$UP_{3}$	4	(16,32)	$\alpha P_{tx}$
$UP_{1}$	4	(16,32)	$P_{tx}$
**CW Assisted Node Prioritization**			
**UP**	**No. of Nodes**	$\mathrm{CW}\in (C_{\min},C_{\max})$	**Transmission Power**
$UP_{5}$	4	(4,8)	$P_{tx}$
$UP_{3}$	4	(8,16)	$P_{tx}$
$UP_{1}$	4	(16,32)	$P_{tx}$
**Normal Network**			
**UP**	**No. of Nodes**	$\mathrm{CW}\in (C_{\min},C_{\max})$	**Transmission Power**
$UP_{1}$	12	(16,32)	$P_{tx}$

**Table 3:** List of notations.

Parameter	Description
$T_{p}$	Transmission Preamble Time
$R_{s}$	Preamble Transmit symbol rate
$T_{phy}$	Physical header transmission time
$R_{phy}$	Physical header rate
$R_{data}$	Transmission data rate
$T_{slot}$	Slot time
$T_{sifs}$	Short inter-frame spacing time
$T_{data}$	Data transmission time
$T_{SB}$	Average backoff time for successfully transmitted packet
$T_{C}$	Average collision time
$T_{TX}$	Average time for single packet transmission
$T_{D}$	Average time for a packet to be dropped
$T_{DB}$	Average backoff time for a dropped packet
$T_{S}$	Average service time
$T_{ACK}$	Average time for acknowledgement of Successful packet transmission
MHR	MAC header in bits
FTR	MAC footer in bits
*τ*	Propagation delay
*ρ*	Bandwidth efficiency
$P_{tx}$	Transmission power
*X*	Payload size
$x^{{}^{\prime }}$	Payload present in block acknowledgement block
$P_{idle}$	idol power
$N_{C}$	Average number of collisions for a successfully transmitted packet
$P_{S}$	Probability for a successful packet transmission
$E_{D}$	Average energy consumption of a node for a failed packet transmiion
$E_{DB}$	Average energy consumption in backoff of dropped packet
$E_{TX}$	Average energy consumption in transmission of a packet
$E_{DC}$	Average energy consumption in collision for a dropped packet
$E_{S}$	Average energy consumption in successful transmission of a packet
$E_{SB}$	Average energy consumption in backoff of a successfully transmitted packet
*R*	Maximum allowed retransmission of a packet
$E_{B}$	Initial energy of battery

**Table 4:** Simulation parameters.

$P_{TX}$	0.8 mW
*α*	1.1
*β*	1.2
*λ*	1.25
Carrierfrequency	$2.42\times 10^{9}$ Hz
$T_{RX-TX}$	0.00021 s
$T_{RX-TX}$	0.00021 s
*X*	2000 bits
Queue length	5
MHR	56 bits
FTR	16 bits
$T_{Slot}$	0.00035 s
*R*	7
$R_{data}$	200 kbps
Beacon	15 bytes
$T_{\rho sifs}$	50 μS
Ack size	9 bytes
Simulation time	30 s
Battery capacity	560 mAH
Superfame length	1 s
*τ*	75 μS
